# Visually representing total pain at the end of life through photovoice

**DOI:** 10.3389/fsoc.2026.1831930

**Published:** 2026-07-10

**Authors:** Naomi Richards, Sam Quinn

**Affiliations:** School of Social and Environmental Sustainability, College of Social Science, University of Glasgow, Glasgow, United Kingdom

**Keywords:** dying, end of life, photography, photovoice, poverty, social determinants of health, suffering, total pain

## Abstract

**Introduction:**

The concept of ‘total pain’ was conceived and utilised by Cicely Saunders, pioneer of the modern hospice movement, in the mid-1960s to highlight to doctors the multidimensional – physical, social, emotional, and spiritual – nature of suffering experienced specifically at the end of life. Photovoice is a participatory visual research method which places cameras in the hands of research participants to document aspects of their own lives – including experiences of suffering - in order to promote critical dialogue with researchers and to reach and influence decision-makers. In this article, we explore connections and overlap between the photovoice method and its outputs, and Cicely Saunders’ concept of ‘total pain’ and her application of the concept.

**Methods:**

We interpretively analysed photovoice data - participant-produced photographs, transcripts of interviews, and other forms of written communication - from two specific participants who took part in a four-year UK-based research study called Dying in the Margins (2019-23). The aim of the study was to uncover barriers to, and experiences of, home dying for people experiencing poverty and deprivation.

**Results:**

The visual and textual data highlight the multiple causes of the women’s suffering, stemming from their housing, financial, material, and relational circumstances.

**Discussion:**

We identify significant alignment between the intentions and outputs of photovoice and Cicely Saunders’ concept of ‘total pain’, which has been foundational to the historical development of palliative care. We argue that both the photovoice method and Saunders’ concept aim to draw attention to causes of suffering beyond physical symptoms, so often the exclusive focus of the clinical encounter. Photovoice images zoom in on the fine-grained intimate details of people’s embodied experience and their environments in much the same way that Saunders presented individual details or fragments of her patients’ stories in order to give substance to, and make real, her concept of ‘total pain’. Both photovoice imagery and Saunders’ elaboration of ‘total pain’ promote emotional identification in onlookers and listeners and thereby become a rallying call for action to mitigate the social determinants of dying. We advocate for further use of photovoice in end-of-life care research, specifically in social and material contexts which are seldom given consideration by policymakers or referred to in housing, public health or end-of-life care policies.

## Introduction

On 3rd April 2023, two women, Stacey and Liz, were dying of cancer in adjacent rooms in a hospice. Research Associate Sam Quinn was visiting the hospice in Glasgow to interview Liz for the final time. During this visit, Sam was surprised to find that Stacey was being cared for in the next-door room. Stacey was heavily sedated by this time, surrounded by tubes and machines, and Sam was unable to speak to her. Stacey’s mother and husband stayed with her around the clock during these final weeks. Stacey and Liz had never met one another, but had both been part of our research study—Dying in the Margins (2019–2023)—focused on barriers to, and experiences of, home dying for people experiencing poverty and deprivation in the UK. Aged 73, Liz was our oldest participant, whilst Stacey was one of the youngest, aged 39. Of all our study participants, Stacey and Liz were the most enthusiastic adopters of one of our visual methods: Photovoice. Both had avidly documented, through photographs, their last months of life.

In this article, we reproduce a selection of photovoice images taken by these two women, interspersed with a selection of their direct quotes and other forms of written communication sent to the research team, in order to reflect on the multidimensional nature of their end-of-life suffering. Stacey and Liz were their real names—used here with their (documented) permission, and indeed at their insistent request.

Our analytical aim is to take up Rowley et al.’s (2021) recommendation to explore the conceptual overlap between Cicely Saunders’ concept of ‘total pain’ and the social determinants of health as they manifest at the end of life, also referred to as ‘the social determinants of dying’ (Sallnow et al., 2022). Saunders (1964) concept of ‘total pain’ emphasises the indivisible nature of the physical, emotional, social and spiritual dimensions of suffering experienced at the end of life and encourages clinicians to treat patients in a holistic way, rather than focusing exclusively on their physical symptoms. As Wood (2025) explains in his book-length genealogy of Saunders’ concept, ‘total pain’ at its core is intended to remind professionals that they need to consider the context of a patient’s wider life; that a patient’s pain extends *beyond* their body to their social network and can be exacerbated by a complex biography, the emotional impact of a terminal diagnosis, and the resurfacing of trauma from across the life course. Since the 1960s when Saunders’ originated the concept of ‘total pain’, awareness of the effects of trauma on people’s bodies and psyches has developed significantly, as has clinical awareness that experiences of poverty and deprivation can increase the likelihood of exposure to adverse and traumatic events (Delgadillo and Richardson, 2025; Lacey et al., 2022).

We are not the first to use the concept of ‘total pain’ to highlight the impact that structural marginalisation has on end-of-life experience, and the need for care which takes greater account of the structural and historical sources of pain shared by certain groups of people or located in more deprived areas (Gunaratnam, 2012). Indeed, in this special issue, Graven et al. (2023) lean on ‘total pain’ to illuminate the specific end-of-life suffering experienced by social marginalised Greenlanders living in Denmark.

In this article, we use Saunders’ (1964) concept as a lens to interpret Stacey and Liz’s photographs and the narratives surrounding them, as well as to think through the value of the images and their accompanying narratives for education and advocacy purposes. We make a claim for photovoice as a ‘tool for action’ (Wang, 1999) which can evocatively present the insider view of the everyday injustices and material deprivations which are experienced both within, but more importantly *outside* of institutions, and therefore beyond what is observable in brief clinical encounters. The women’s photographs and narratives show that the social determinants of dying are more than simply ‘background social context’ which it would be nice to know. These social determinants are indivisible from the physical pain and therefore fundamentally constitutive of how dying is experienced.

## Materials and methods

Photovoice is a participatory research method which places cameras in the hands of research participants so they can record scenes, objects and moments that represent their experiences and priorities. The method was pioneered in the early 1990s by Wang and Burris (1997) to: (1) empower participants to document their ‘strengths and concerns’ through the taking of their own photographs; (2) promote critical dialogue and knowledge through discussion of their photographs; and (3) reach policymakers. It is often conflated with photo elicitation but is distinct from that method because of the crucial third stage in the process — reaching policymakers. Photovoice is a widely used research method with a significant body of literature supporting its practice and evidencing its effectiveness (Catalani and Minkler, 2008).

The photovoice method is inductive and involves an iterative cycle of research, discussion and action (Milne and Muir, 2020). Participants can see, interpret, and appraise their images as they generate them, discussing the process either collectively in a group or one-on-one with the researcher. These discussions are recorded and form data which are analysed alongside the visual data (Milne and Muir, 2020). The process encourages reflection, sparks dialogue between the participant and the researcher and generates data that combines photographs with spoken explanation — involving expansion, contextualisation, and the making of connections. In other words, the combination of participant-produced images and words enables the rich layering of stories and meaning (Richards et al., 2023). In the third and final step of the process the images need, one way or another, to reach policymakers. This is commonly achieved through an exhibition, where the photographs are displayed accompanied by short text narratives (Milne and Muir, 2020). The literature reports the following potential benefits for participants who engage with the photovoice method: enhanced self-esteem, confidence and control (Teti et al., 2013); the raising of participants’ critical consciousness (Carlson et al., 2006); and the facilitation of creative expression and meaning-making (Tishelman et al., 2016).

There is an established body of literature supporting the use of photovoice with structurally marginalised groups (e.g., Liebenberg, 2018; Loignon et al., 2023) which underpinned our rationale for using the method. There are also a handful of examples of the method being used in palliative and end-of-life care research, notably Tishelman et al.’s (2016) and Hajradinovic et al.’s (2018) studies in Sweden and Moore et al.’s (2013) in the UK. Elsewhere, we have argued that photovoice holds significant potential in end-of-life research to capture the smaller, everyday and cumulative aspects of suffering that interviews might miss (Quinn and Richards, 2025b).

In Dying in the Margins, we provided participants with simple digital cameras and audio recorders and invited them to capture anything important to them in their everyday lives. Several participants opted to use their phone cameras instead. The study was undertaken against the backdrop of the COVID-19 pandemic, but as restrictions were relaxed, we held periodic touchpoints – once every few months depending on participant preference — to download photographs and discuss them, treating the resulting images and conversations as a single analytic data set (Quinn and Richards, 2025b). These touchpoint interviews were transcribed verbatim. Embodying the spirit of the photovoice method, we respected participants’ agency and exercised flexibility in how participants shared their data. The involvement and preferences of each participant varied (for more detailed reporting of our use of the method, see Richards et al., 2023).

Once data collection ended, three members of the Dying in the Margins research team analysed the single analytic data sets — all images, textual data, and audio data for each participant in four in-person “research retreats” (Tishelman et al., 2016) held over an 8 month period. In some photovoice studies participants are fully involved in the analysis and dissemination stages, but this was not possible in Dying in the Margins as participants were too ill or had died. The team used interpretive visual analysis (Riessman, 2008) which involved asking questions of the data such as: What is the manifest content of the image versus its symbolic meaning? What tangible and intangible aspects of suffering are represented? These questions and more (see Richards et al., 2024) prompted in-depth discussion as both the individual and cross-cutting themes were drawn out. Notes were taken by Richards during the sessions, which were also recorded for later cross-checking. All data (both visual and textual) were imported into the software programme NVivo and coded by Richards. Coded data for Stacey and Liz were used as the basis for this article. This dynamic interpretive process of analysis informed the curation and construction of an exhibition called The Cost of Dying which ultimately allowed us to sense-check our interpretation with stakeholders and the public via solicited exhibition feedback (Quinn and Richards, 2025a).

In this article, we have chosen to focus on two participants from the study: Stacey and Liz. The reason for their selection is twofold. First, in our estimation and from following them over several months, these two women experienced a multifaceted form of distress which aligned to Saunders’ conception of ‘total pain’. Second, both participants embraced the photovoice method thereby providing a substantial visual record of their last year of life. Stacey shared 80 photographs in total, which she preferred to send via WhatsApp with accompanying text commentary; only two in-person touchpoints were held. Liz shared 150 photographs in total over eight touchpoints, preferring in-person meetings at a café in her local library. Both participants gave informed consent not only to participate in the study but to the use of their real names, photographs and relevant personal details being used in publications and for public dissemination.

We aim to maximise use of Stacey and Liz’s photovoice imagery within the space allowed in this article. The photographs and quotes have been chosen and sequenced by us to tell their stories in a way which, in our estimation, is likely to: (1) best capture the reader’s imagination; (2) signal to readers the wider socio-economic and relational context of each woman’s life; and (3) stay true to their stories as they were told to us. We are cognizant of the fact that images are polysemic and can be read in multiple ways by different audiences (Barthes, 1977). Our interpretive framing is intended to enhance people’s ability to ‘read’ the photographs: to gain a sense of both what the participant was trying to show and what kind of wider social circumstances and experiences the image references. However, we do not intend, nor would it be possible, to foreclose readers’ own interpretation of the images and in this sense the article is one of visual provocation, opening up discussion about the representation of end-of-life suffering as much as revealing ‘hidden’ end-of-life suffering.

We recognise that photovoice in end-of-life research raises ongoing ethical and representational challenges, particularly where images of dying, and of financial and material hardship may expose participants to stigma or misrecognition. In line with our previous reflections on the method, we treated consent as an iterative process and prioritised Stacey and Liz’s agency, including respecting their choice to be seen and named (Richards et al., 2023).

## Results

### Stacey

“Ten years […] 10 years fighting this.”

Stacey, age 39 when she died, was diagnosed with a rare genetic condition called Li-Fraumeni-syndrome 10 years prior to being referred to our study. Li-Fraumeni syndrome increases a person’s susceptibility to cancer such that, across her relatively short life, Stacey had experienced cancers in her leg, breast, lymph nodes, back, heart, liver and brain.

**Figure 1:**
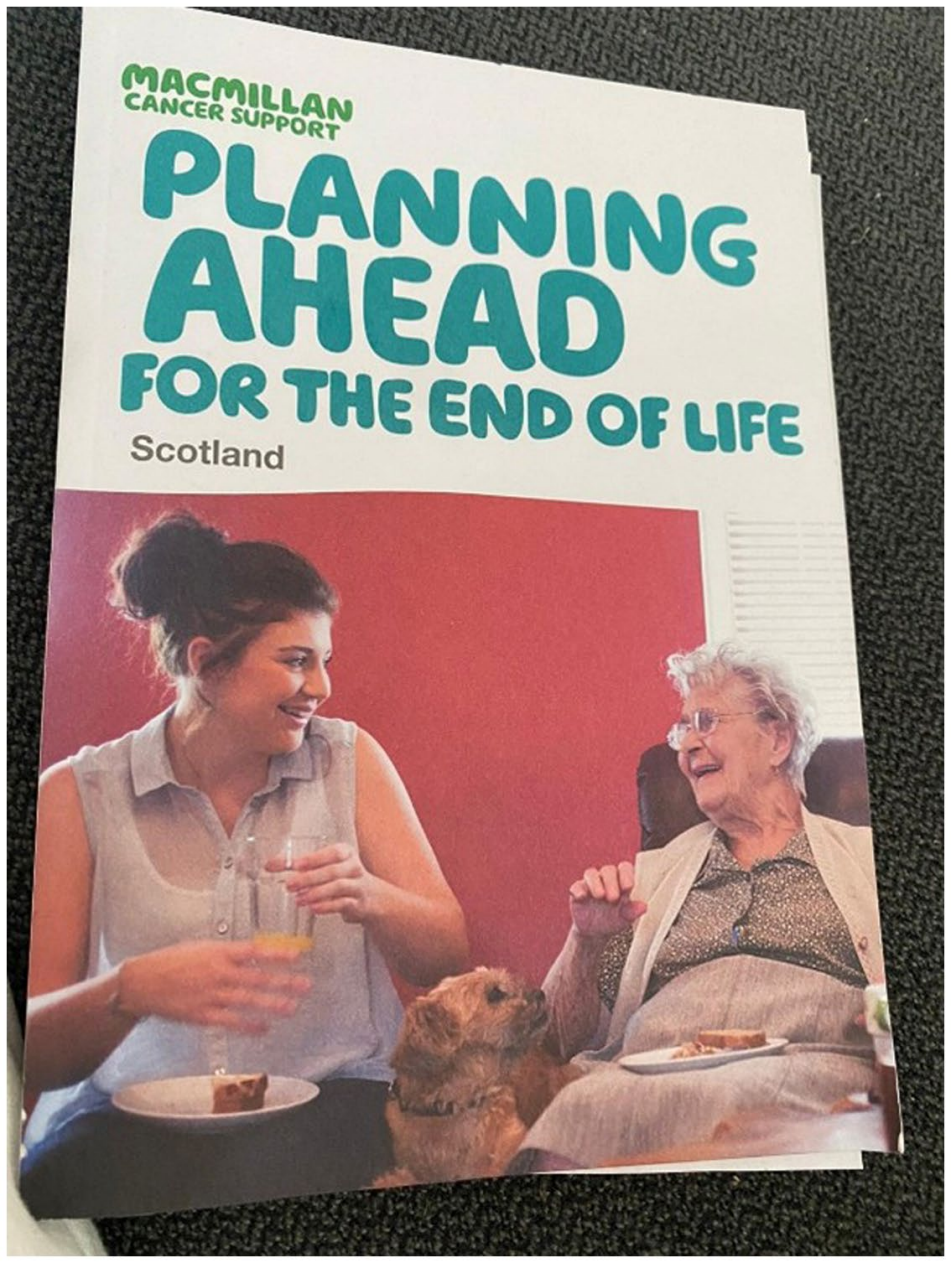
Stacey’s photovoice image of a Planning Ahead document she was sent by Macmillan Cancer Support ©Dying in the Margins 2022 all rights reserved.

Stacey lived with significant uncertainty which in itself caused her distress: “They said that they don’t know what’s going to happen with me which is really scary.” We could see from her photovoice imagery (Figure 1) that terminal illness charity Macmillan Cancer Support had attempted to initiate advanced care planning conversations via a leaflet which had arrived in the post during one of the COVID-19 lockdowns. But this impersonal intervention was not well received by Stacey or her family, perhaps because it was insensitively handled via an impersonal leaflet through the post, or perhaps because it came at the wrong time in relation to her awareness of the fact that she was dying:

I do not know why they do it [post the leaflets]. Do you not need to give people hope?

In fact, for quite a large proportion of her time in the study, Stacey was focused on seeking a cure for her illness, and through increasingly drastic means. However, as time went on her comments revealed an increasing ambivalence—continuing to seek a cure whilst also speaking openly about “dying” and explicitly naming it. We perceived in her comments a gradual shift in focus to just wanting her suffering to come to an end.

Although Stacey was seemingly affronted by the leaflet which arrived through the letterbox, she did tell Sam that she would like to talk to someone about what she was going through:

It would be good to just have somebody to pick up the phone and talk to. I do not know, the Samaritans or something. Do the Samaritans still exist?

But what was offered to her by the services she was in contact with felt bureaucratic and formulaic rather than person-centred:

There was a guy and he was just reading off a checklist or something, that’s what it felt like.

Stacey had lived in her flat for 14 years; she had been a hairdresser and worked at a perfume counter. According to her husband, Joost, when Stacey could not work for a few months following platinum chemotherapy, the most potent available, she was dismissed because she was on a zero-hour contract. This left her without money for heating and “the house so cold that when she awoke, her glass of water was frozen on her bedside table” (Glasgow End of Life Studies Blog, 2023b). Stacey told Sam about the damp in the flat and the black mould which was growing on the windows.

**Figure 2:**
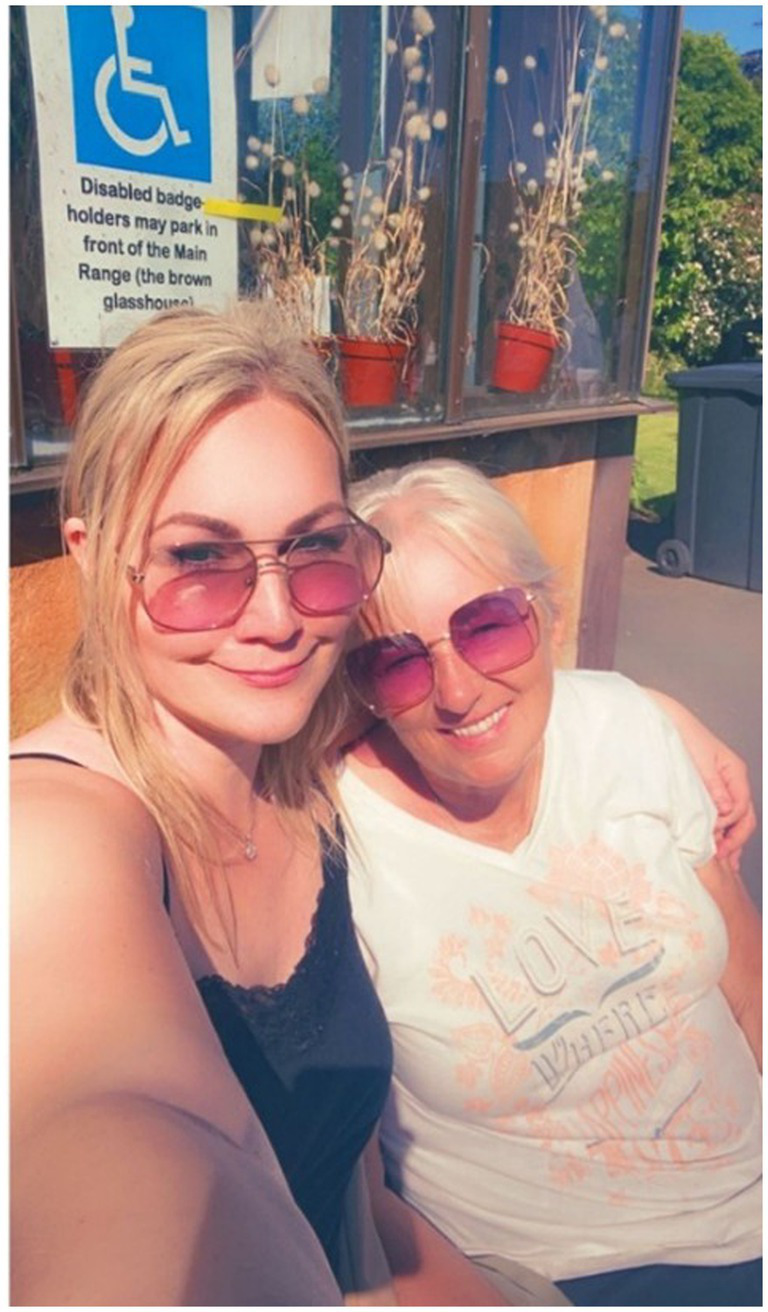
Stacey’s photovoice image of her with her mum Irene who had moved in to help take care of her ©Dying in the Margins 2022 all rights reserved.

**Figure 3:**
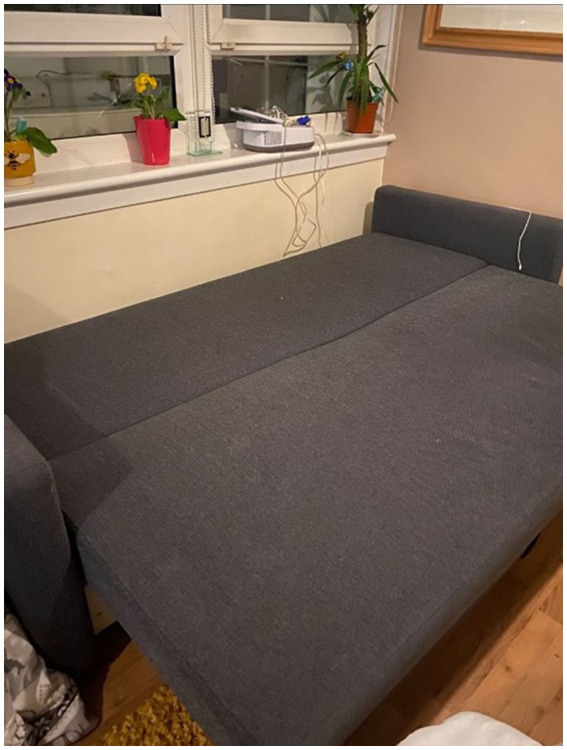
Stacey’s photovoice image of the sofabed which her mum slept on, and which took up the living space ©Dying in the Margins 2022 all rights reserved.

Joost was a Dutch national and worked away from home for long stretches of time. Stacey’s mother, Irene, had moved in to provide essential care for her when Joost was away (Figure 2). When Stacey joined the study, Irene was sleeping on the sofa bed which made the one-bed, sixth-floor flat feel cramped for the three of them (Figure 3). Stacey was also affected by the noise pollution from living in close confines with others in her busy tower block, as well as by the lack of access to outside space. Her block of flats was situated in the middle of a built-up inner-city area with no green space or seating areas in the vicinity. Stacey’s situation was indicative of socioeconomic inequalities in access to green and blue space across the UK (Public Health Scotland, 2026):

I cannot go outside. There’s nowhere for me to sit. There’s no spare bedroom. I’m just trapped in this.

This feeling of being “trapped” led Stacey to become preoccupied with moving; a preoccupation which grew more intense as her cancer advanced and her symptoms worsened:

I do not want to be in here. Every morning, they have the diggers digging the road and I’ve got three brain tumours. They’re going on every single morning (…) I do not want to be here in this house.

**Figure 4:**
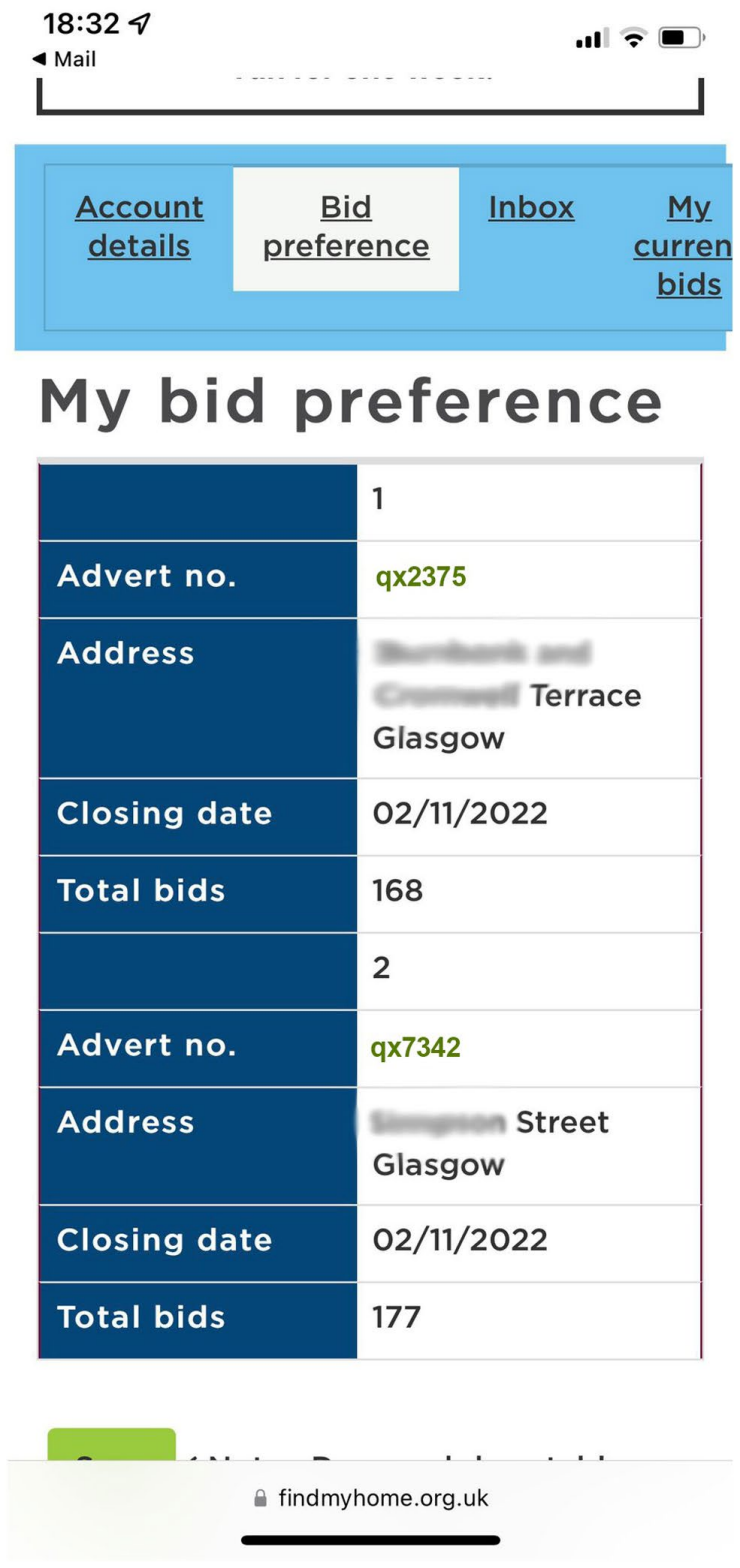
Stacey’s photovoice image of the bid she placed on another flat, with the ‘number of bids’ on the same housing association property listed at 177 ©Dying in the Margins 2022 all rights reserved.

When Stacey was well enough to self-advocate, she repeatedly bid on flats—“I’ve been phoning and phoning constantly trying to get out of here”—but found that there was intense competition and was told by her housing association “there’s no houses”. Figure 4 shows an email from the council notifying Stacey that her bid was the 177th placed on the property.

**Figure 5:**
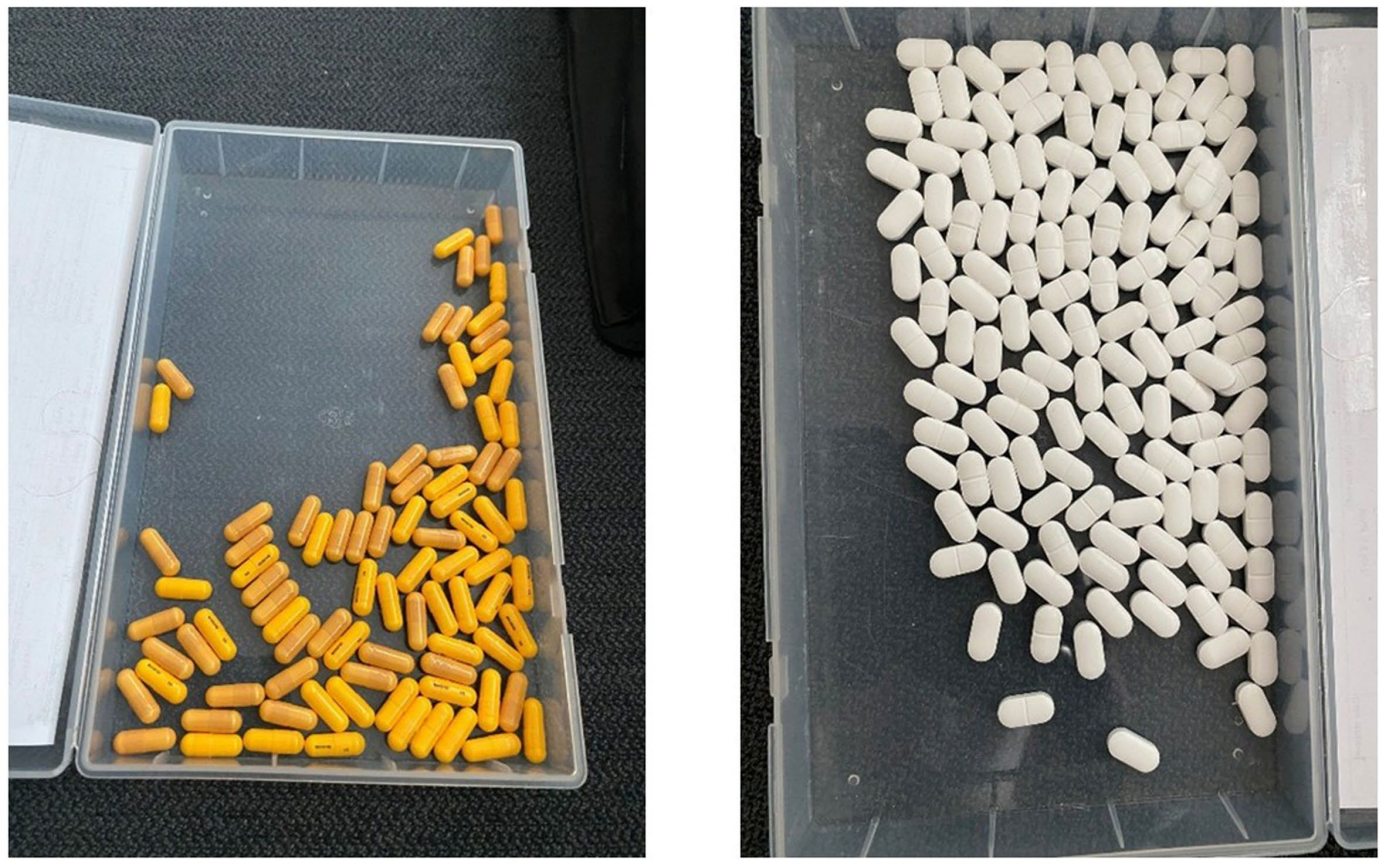
Stacey’s photovoice images of the pills she had to take for her cancer and her pain – “just feel like I’m choking on them all” ©Dying in the Margins 2022 all rights reserved.

As Stacey “battled on” in her words, trying to move somewhere more suitable, her physical symptoms were becoming more painful and debilitating, requiring a heavy medication regime, as her photos revealed (Figure 5):

In the morning I’m taking them all and then by lunchtime I just feel like I’m choking on them all.

**Figure 6:**
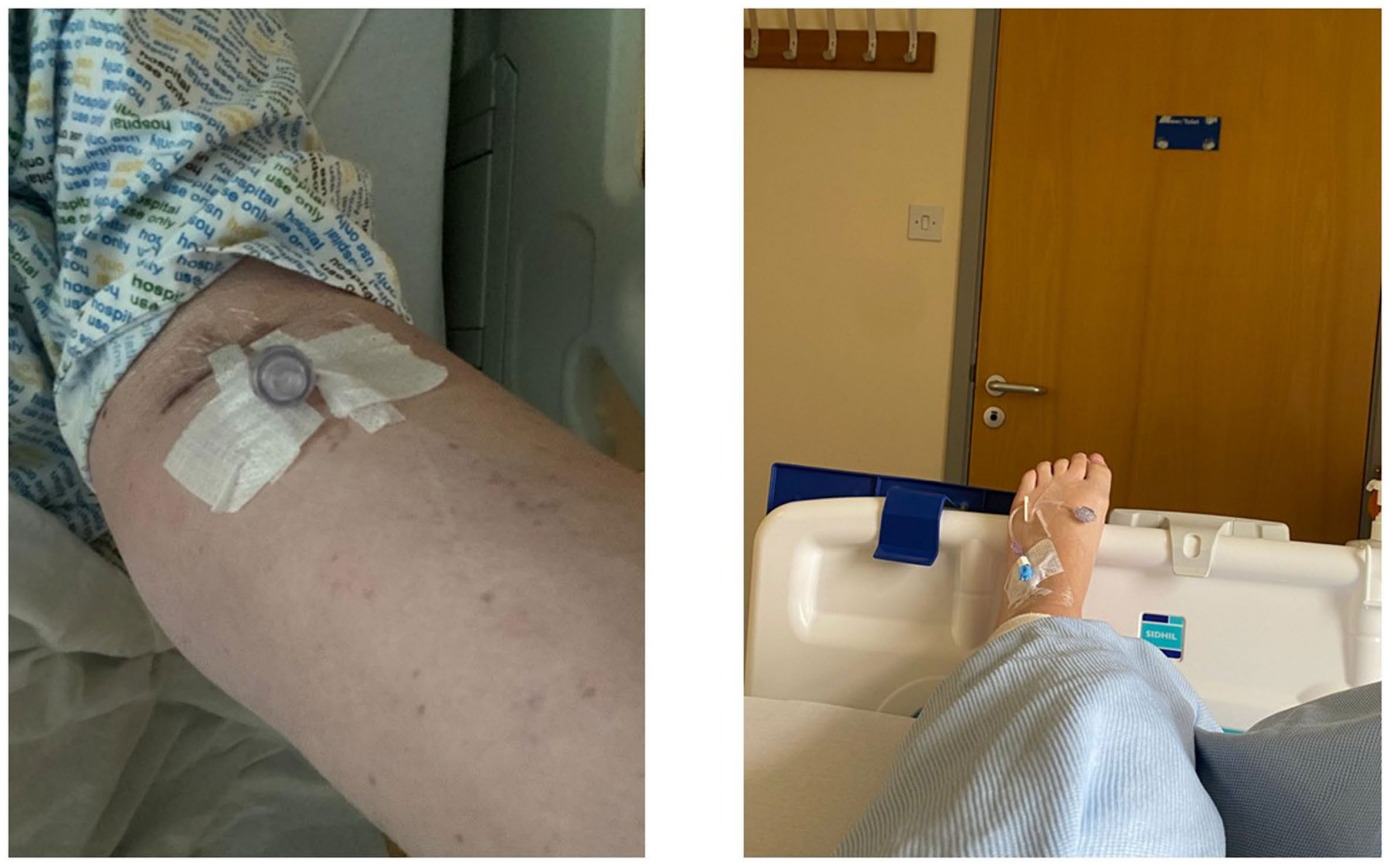
Stacey’s photovoice images of her IV lines – in her arm and her foot – “It’s just constant needles, constant drugs” ©Dying in the Margins 2023 all rights reserved.

There were frequent trips to hospital and Stacey took close up photographs of what was being done to her body on these visits (Figure 6). Her images reveal the unrelenting nature of the medicalisation of her body:

It’s just constant needles, constant drugs. It’s awful, constant machines.

**Figure 7:**
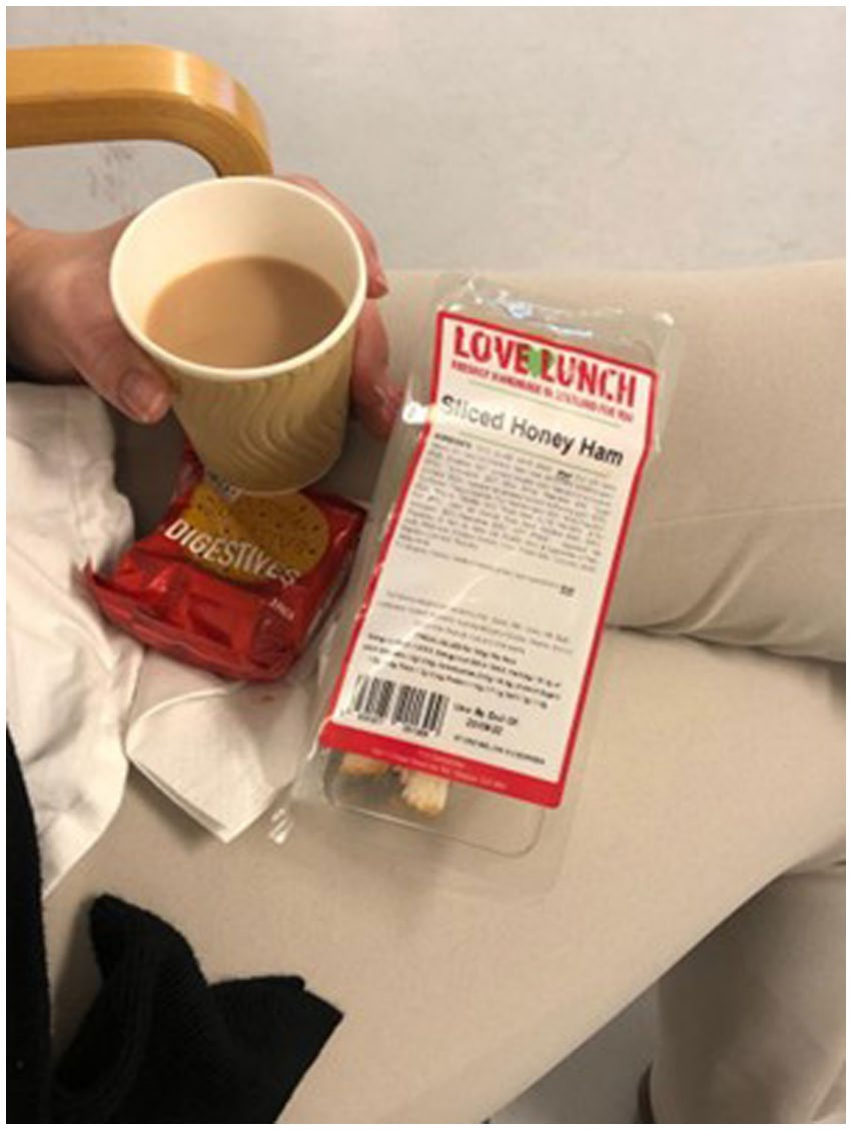
Stacey’s photovoice image of the cup of tea, sandwich and biscuits provided by “wee volunteer women” on her long stays in hospital ©Dying in the Margins 2022 all rights. reserved.

Spending so much time in hospitals entailed a lot of waiting around, sometimes accompanied by her mum, sometimes on her own. The photograph Stacey took of the cup of tea, sandwich and biscuits delivered by volunteers as she waited between appointments in the hospital (Figure 7) represents the everyday small acts of kindness she encountered and can be viewed as a visual counterpoint to her many photographs of “constant needles, constant drugs.” Photographs like these record mundane occurrences and interactions which punctuate life with a terminal illness and might not be picked up by other research methods as they may well be considered too mundane to mention in an interview (Sweetman, 2009).

Best thing about this ward is the wee volunteer women coming round with tea and sandwiches and biscuits.

As Stacey’s physical suffering and medical surveillance increased, so did her dependency on her husband and her mother, generating new forms of relationality:

My poor partner is run ragged with me and my family have had to do so much for me I have not been able to dress myself or shower or bath for 6 months myself without help as I’ve fallen out the shower twice and cannot get out the bath.

Her words express her guilt at needing help, particularly intensive physical care. Some months into Stacey’s involvement in the study, Joost received an unexpected diagnosis for a serious health issue of his own, requiring an operation with a long recovery. This meant that his ability to care for Stacey was compromised:

I do not know who’s providing care for who.

Joost’s operation was successful, but the additional stress was clear in her images and written communication.

**Figure 8:**
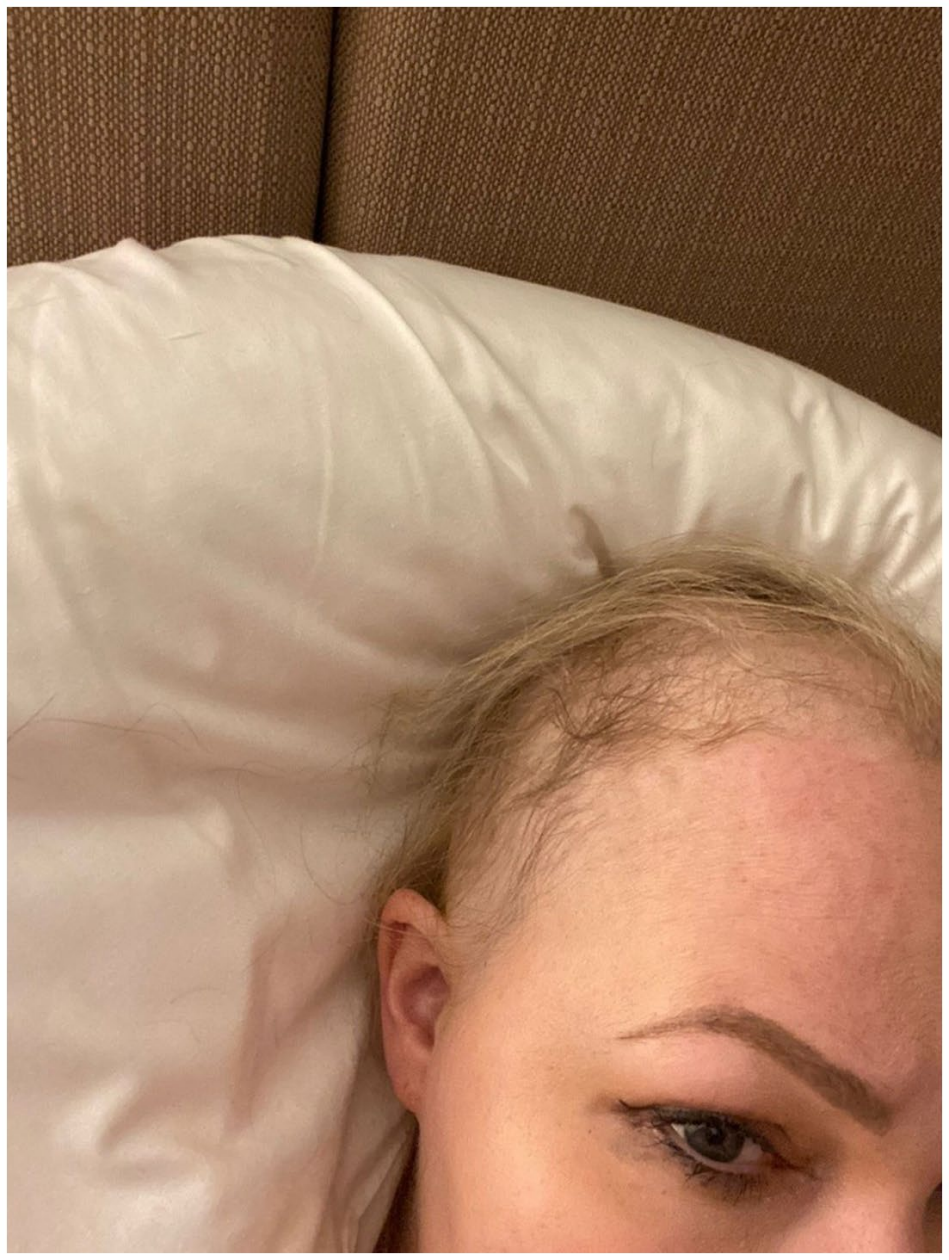
Stacey’s photovoice image of her hair loss caused by her chemotherapy which as a former hairdresser she found very distressing ©Dying in the Margins 2022 all rights reserved.

Stacey continued to press for a new flat for them to move into but with no luck. Her photographs show that she was physically declining all the time. She felt unprepared for losing her hair from the palliative chemotherapy (Figure 8) and she felt that her distress—as a former hairdresser and as a relatively young woman—was not recognised by the hospital staff:

… they think that’s nothing, it’s just part of the symptoms but it means an awful lot to an awful lot of people […] Somebody is sitting devastated because they are going to lose their hair and they get brushed off.

Stacey’s comments from this time reveal that her existential suffering was intertwined with her physical suffering. When her cancer spread to her brain, she began to reckon with the imminence of death:

I just feel close to death now since this last thing because it’s my brain and it’s messing with my brain and messing with my body. I cannot do things. I cannot stand. I’m just… it’s taken over me, the illness. I just feel like it’s taken over me.

Her expression “it’s taken over me” encapsulates Stacey’s fragility at this point—her ‘total pain’ as a whole overwhelming experience. Just a few weeks before her death, Stacey got the keys to a two-bed, ground floor flat: “I could actually just walk out the front door and be outside getting some fresh air.” However, when she arrived to look round with Joost, she immediately collapsed. She was taken by ambulance to hospital and from there was transferred to the hospice where she remained until her death. After all her requests to move, she never got to spend a night in her new home.

Her final contact with the research team came 2 weeks before her death, not through a text message, but through a comment she made on our blog post about the upcoming photography exhibition (Glasgow End of Life Studies Blog, 2023a):

I would like to dye in a lovely house hould.

The illness and medication had taken over at this point, and she was clearly struggling to communicate. Yet her message shows that she was still thinking about wanting to be home, which for her was somewhere offering comfort, peace and quiet, and access to fresh air. Stacey’s dedication to the study and the photos she entrusted to us documenting her fight for housing, her gruelling medical regime, and her determination to search for a cure until every avenue had been exhausted, showed her ability to self-advocate until she was very near to death. We read her photovoice as a form of visual activism, to share with a wider audience aimed at social landlords, clinicians, policymakers and politicians. In Stacey’s words:

Maybe it will flag up what’s happening, that it’s not right.

### Liz

Liz was 73 and living with terminal lung cancer when she signed up to take part in the study. Whilst much closer to the average age of death than Stacey, Liz was still below the average for women [81 years in Scotland in 2022–2024 (National Records of Scotland, 2025)]. Like Stacey, Liz lived in a high-rise block of flats, in an area ranked amongst Scotland’s most deprived (Scottish Government, 2020). Life expectancy for women like Liz living in the most deprived areas of Scotland is 10.5 years lower than in the least deprived areas (National Records of Scotland, 2025).

**Figure 9:**
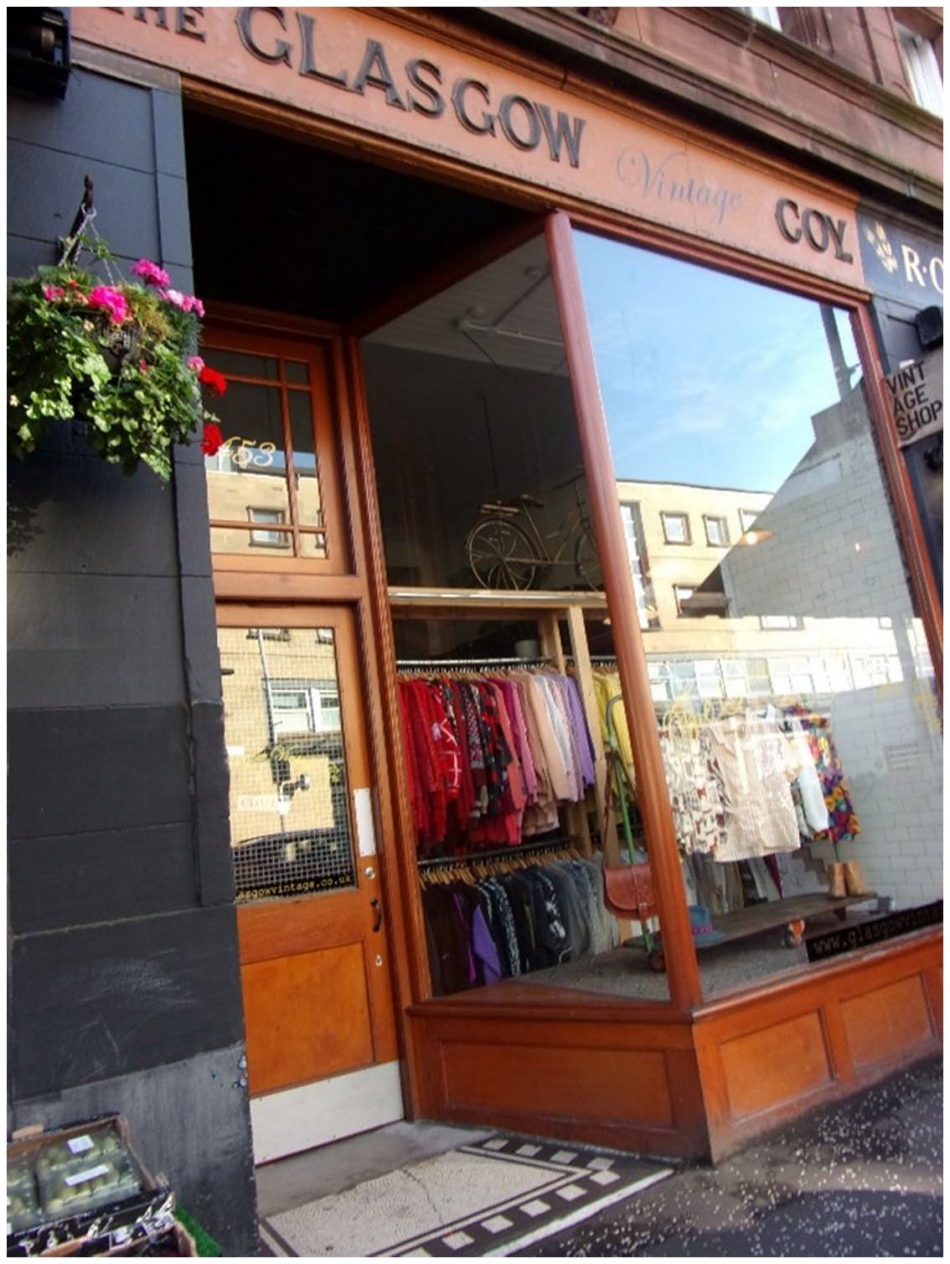
Liz’s photovoice image of one of the vintage shops she frequented in the city, as much to talk to the staff as to buy clothes ©Dying in the Margins 2022 all rights reserved.

**Figure 10:**
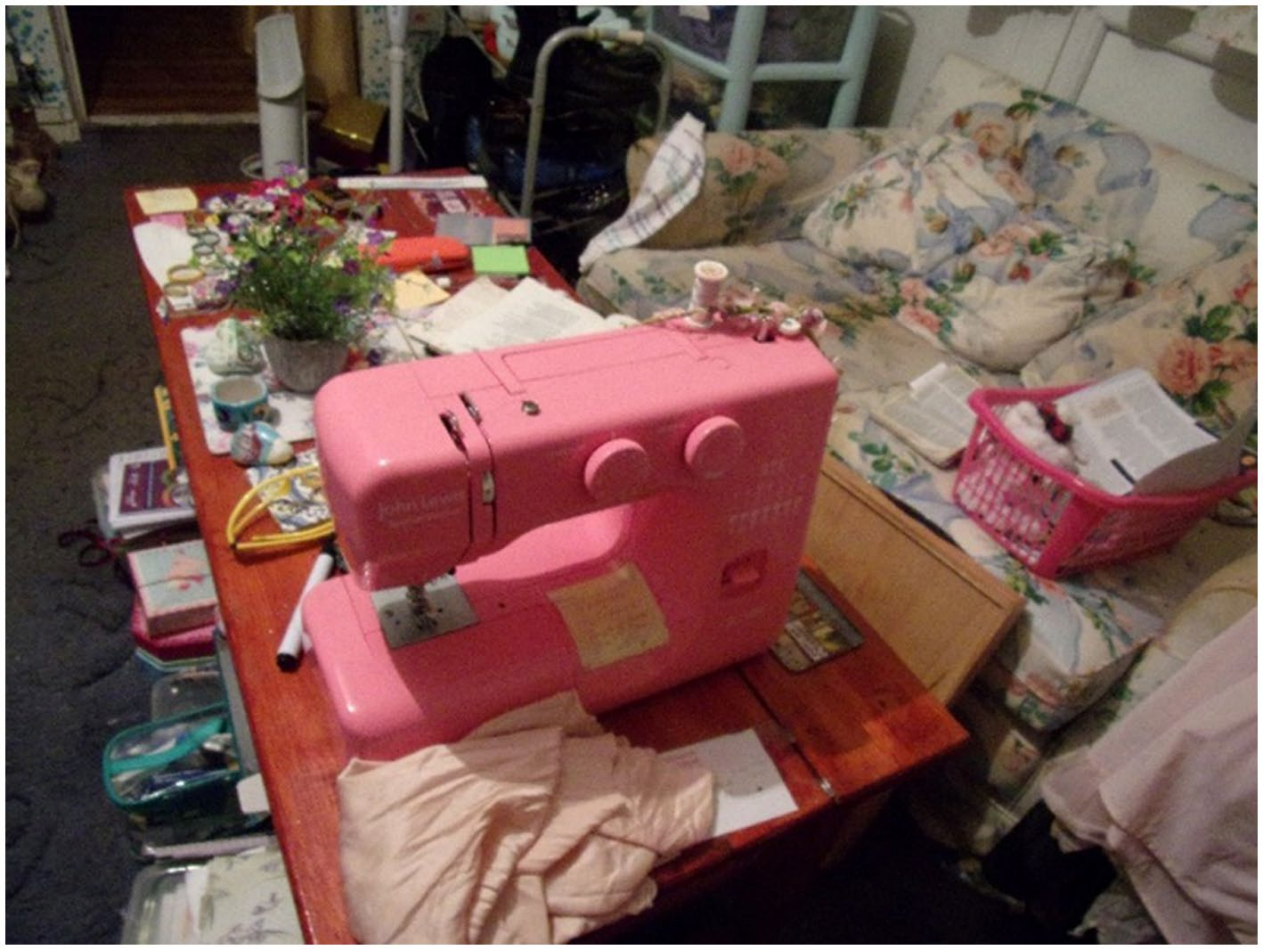
Liz’s photovoice image of her pink sewing machine – she said using it to create clothing brought her comfort ©Dying in the Margins 2022 all rights reserved.

Unlike Stacey, Liz did not take a single selfie of her face. Instead, she began the study by taking her camera on walks around the city [known as a photography walking tour in the literature (Fink, 2012)], to show us the places she liked to frequent and where she felt at home. Liz was a fashion lover and liked to visit charity shops and thrift stores, as much as for the company of talking to the staff as to try on clothes (see Figure 9). Adapting clothing or making new ensembles using her bright pink sewing machine (Figure 10) was an activity which brought her comfort:

If I’m down, I just get through a pile of sewing.

**Figure 11:**
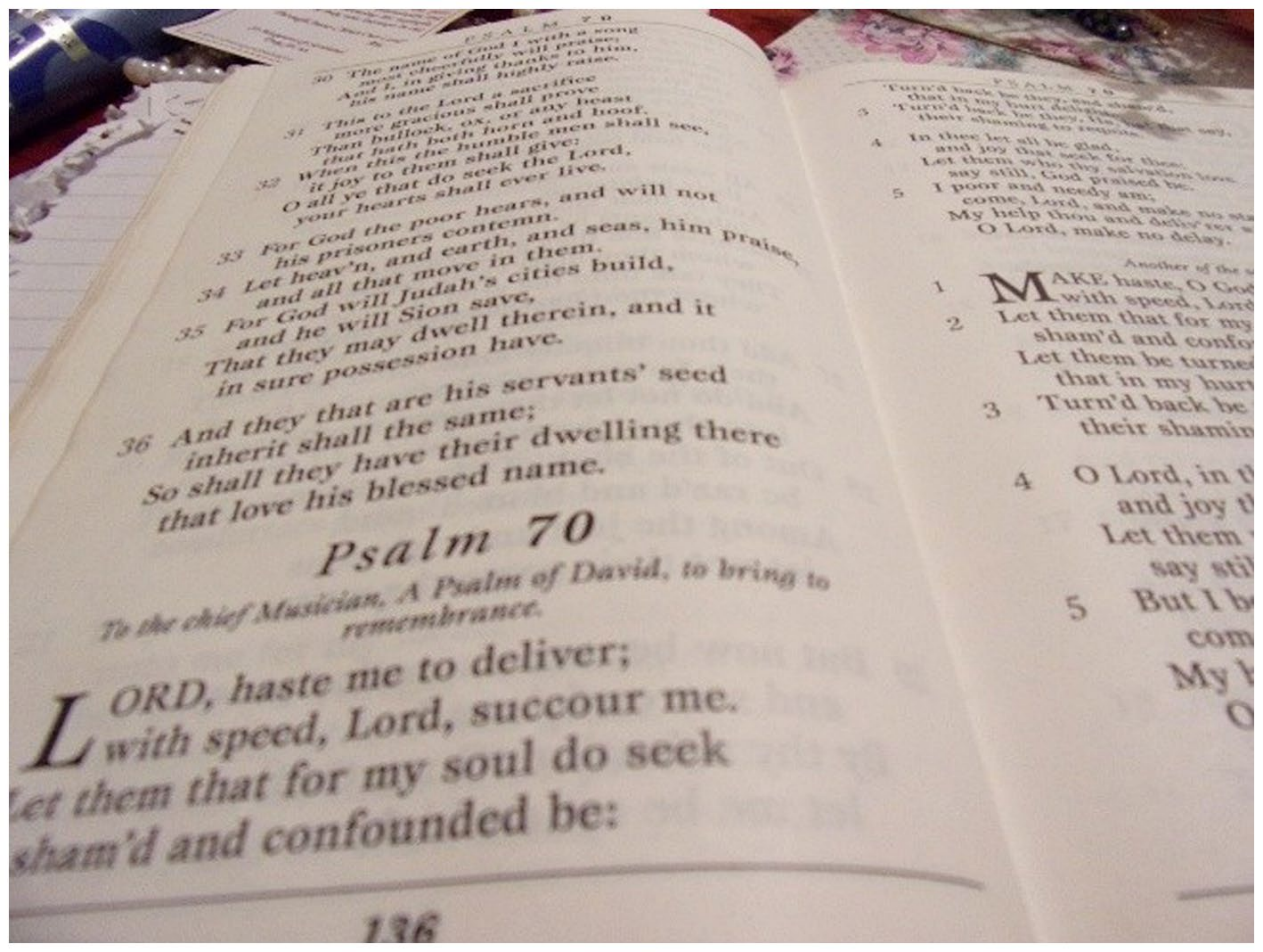
Liz’s photovoice image of a Biblical psalm – she said reading it brought her comfort ©Dying in the Margins 2022 all rights reserved.

Her one-bed flat where she had lived for 20 years was strewn with bright coloured clothes (Figure 11) and a lifetime of mementos. Whilst she recognised that other people might characterise her flat as “cluttered,” to her it was home and where she wanted to remain until the end of her life:

I cannot face being in a hospice with no visitors at all. I can see that happening. And no access to my stuff … I want to remain at home.

Liz was a devout Christian and took photos of the (many) churches she would visit seeking comfort and companionship as well as spiritual solace. She said:

I feel there must be a reason or purpose for the suffering to make sense.

**Figure 12:**
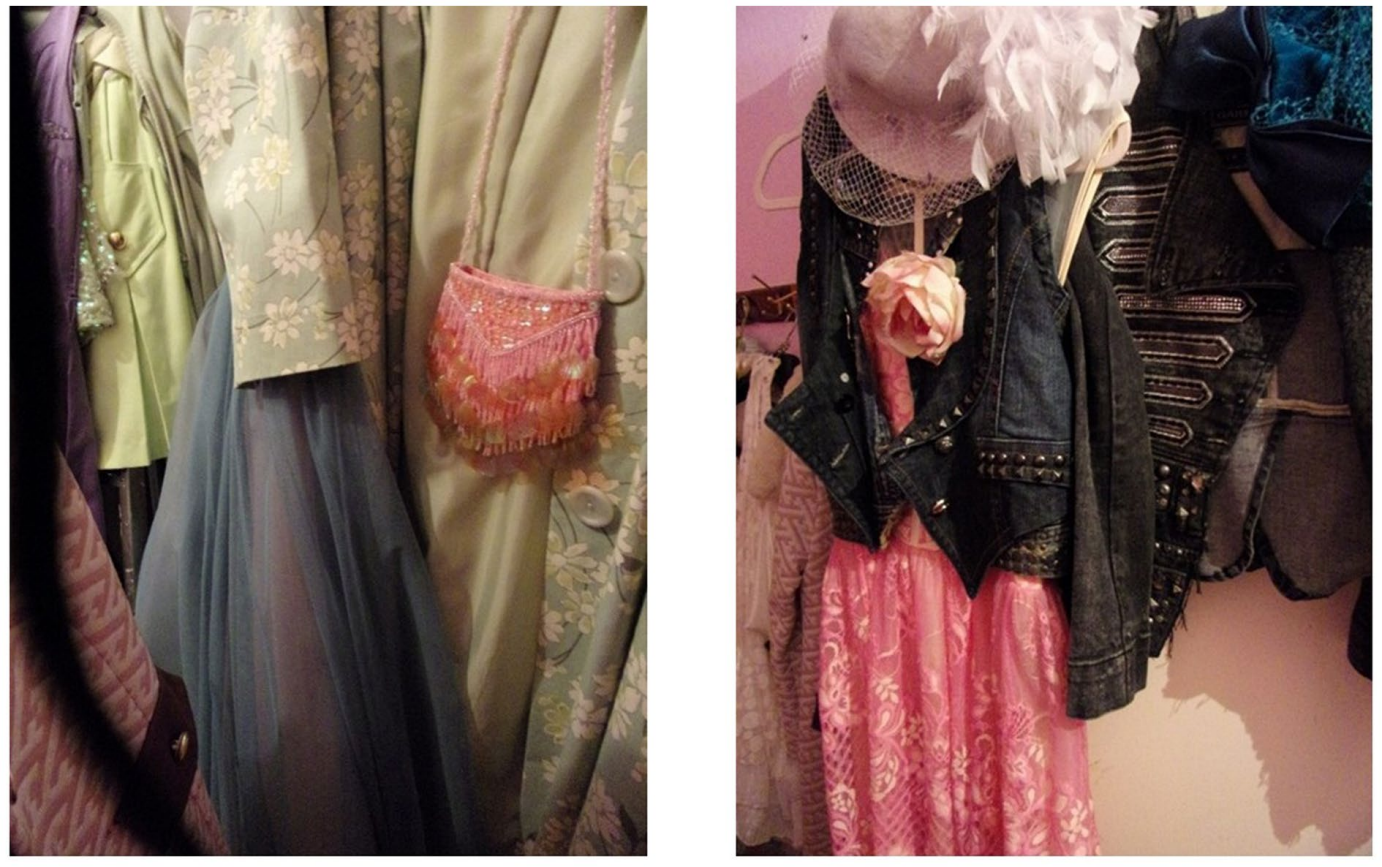
Liz’s photovoice images of her cupboards full of colourful clothing ©Dying in the Margins 2022 all rights reserved.

Liz felt ostrasised by these church communities, which in her account was a result of her partner being transgender: “unfortunately, most churches don’t sit happily with that.” Her photographs convey the many attempts she made to find acceptance and somewhere she could practise her faith alongside others. On days when Liz felt too sick to leave the house, or felt too “judged” by the congregations of these churches, she would seek comfort in revival radio and reading psalms from the Bible (Figure 12):

The radio is a big help to me when I cannot get, or do not feel safe.

**Figure 13:**
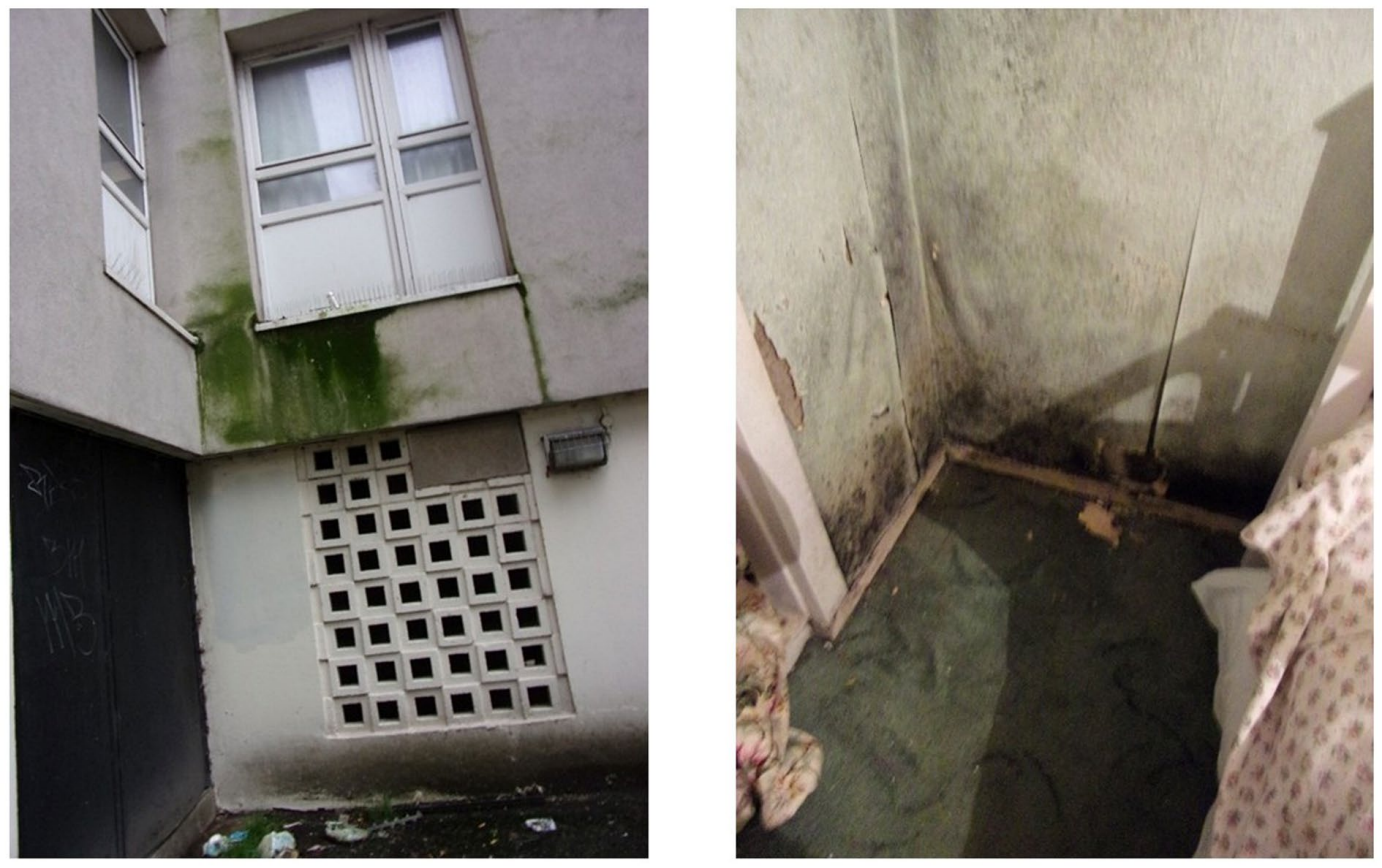
Liz’s photovoice images of the external (left) and internal (right) black mould on her flat ©Dying in the Margins 2022 all rights reserved.

Liz was a board member for her housing association, advocating for other tenants’ rights and trying to get the association to make improvements to the material fabric of the building. But Liz’s own flat was in a poor condition and much of her distress stemmed from the material insecurity she felt when she was inside her own home. Just outside her flat, extensive black mould had taken hold which was now seeping through the wall into her bedroom (Figure 13). Liz was breathing in spores every night. As with Stacey’s situation, the housing association appeared unresponsive to her requests for help. She felt blamed by them for keeping her thermostat low, which she did to keep her heating costs down, telling us:

The housing worker came out and said it was my fault, but it’s not—it’s black outside. They will not listen to me.

Eventually, the housing association power-washed the exterior of the building, but Liz was told she would need to move out to deal with the interior. With so little time left, she could not face the disruption:

How can I move? I will not move […] moving is an upheaval. I could end up somewhere worse.

In addition to the black mould, her flat became flooded when there was a leak in the flat above, and once again she found the housing association unresponsive. We noted a growing sense of despondancy: “there’s so many repairs now.” Like Stacey, Liz was also dealing with noise pollution—in her case from the roadworks outside her flat on a nightly basis. When dealing with housing issues at the very end of her life, Liz perceived that the people working within the system sometimes did not believe her and lacked compassion, despite the fact that she was dying:

It has occurred to me in the last 6 months that people who live in these difficult blocks or areas that come across things like severe and terminal illness, that they are quite honestly considered as a mental health patient.

Whilst on the face of it, considering her mental health might be considered holistic care in line with recognition of her ‘total pain’, when offered antidepressants in isolation without other forms of support, Liz experienced this as a de-legitimisation of her physiological pain and yet another example of being disdisbelieved. Locating the social determinants of health at the level of the individual and their mental health issues or “social problems,” as Liz put it, rather than seeing these determinants working at the level of society, resulted in her feeling stigmatised, and quite often dismissed within encounters with clinicans or housing officials.

As Liz’s condition deteriorated, she struggled to keep on top of routine domestic tasks, and with no practical support on offer, this became a source of shame to her:

It makes me exhausted, but my flat is very dirty, I’m ashamed of that, but there’s nothing I can do […] I’m just so drained.

**Figure 14:**
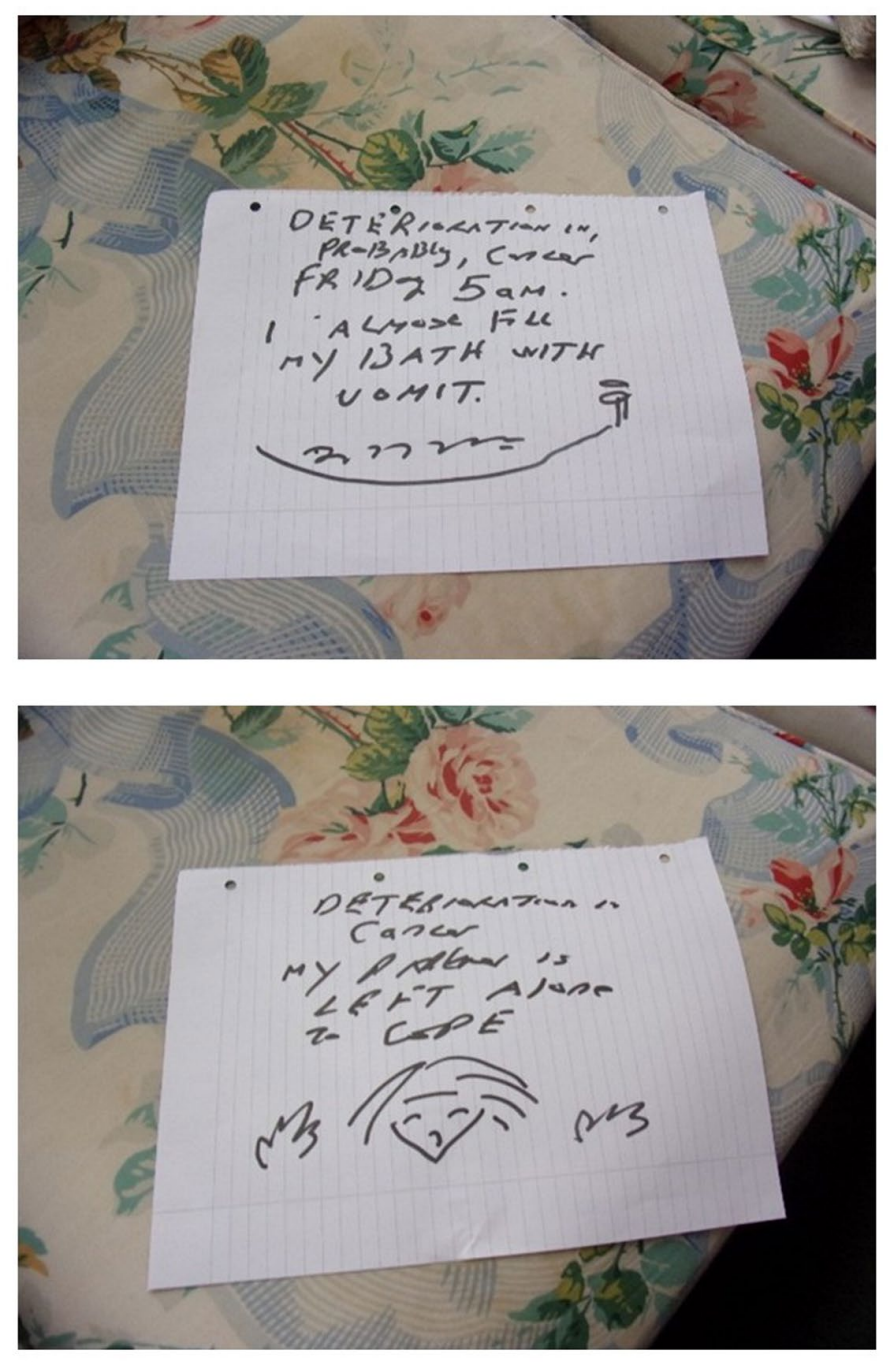
Liz’s photovoice images of the handwritten notes scribbled in the middle of the night which read (top) “Deterioration in, probably, cancer. Friday 5am I almost fill my bath with vomit” (bottom) “Deterioration in cancer. My partner is left alone to cope” ©Dying in the Margins 2022 all rights reserved.

She took photographs of hand-written notes like the ones in Figure 14, often written in the middle of the night. She told Sam:

I’ve got more tired, more fatigued … and I’m starting to vomit up food. I just do not think I can cope. I really cannot.

She began to wonder who would take care of her at the very end, anticipating that her partner would be unable to do so because of their own health issues. Liz lived alone and was estranged from other family members, and in her own words felt a profound sense of loneliness and isolation:

The shock to me—and this is why you are doing the study—is that I’ve come to realise there is no care or support for people like me, living in areas like mine, where my family do not want anything to do with me. I truly do not have a voice. And I was thinking: how hard does this have to get?

**Figure 15:**
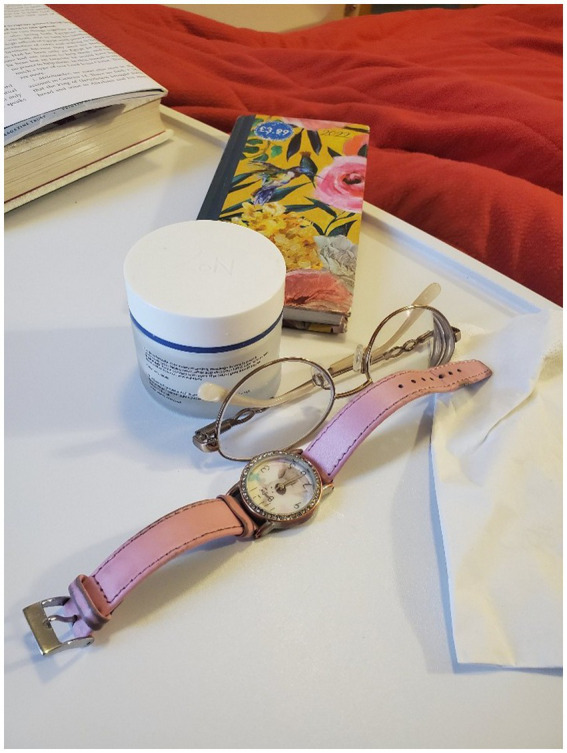
Liz’s photovoice image of the small number of possessions she had with her in the hospice when she died ©Dying in the Margins 2023 all rights reserved.

The photovoice helped Liz to feel a sense of purpose; she wanted to “use it as a voice for people in my situation.” As the repairs to her flat mounted and her physical symptoms increased, the relationship with her partner grew more strained to the extent that she felt she had little choice but to move into the hospice. The care package offered in her own home was just 30 min twice a day which was never likely to sustain her dying at home without family or friends to support her. Although Liz was happy to be looked after in the hospice once she moved there, her anticipation of a lack of visitors was born out, and her meagre belongings (Figure 15) stood in stark contrast to the colour and flamboyancy on display in her flat.

## Discussion

Saunders (1964) pioneered the concept of ‘total pain’ in the 1960s to challenge biomedical reductionism, emphasising instead the indivisible nature of the physical, emotional, social and spiritual dimensions of suffering experienced at the end of life. Whilst multiple interpretations of the concept exist in part because it was ill-defined by its creator, at its root ‘total pain’ was intended to encourage clinicians to treat patients in a more holistic way and ask them questions about, and to take into account, the emotional reality of their lives (Wood, 2022, 2025). Saunders’ intention, according to Wood (2022), was to “reframe” the relationship between medical professionals and their dying patients such that patients’ own “storeys” (as she called them) about their wider life would be attentively listened to and taken seriously. To engage and sustain the attention of her clinical audiences, Saunders would populate her talks and her articles with anecdotes comprised of everyday details about her patients, using the metaphors patients themselves used to describe their pain, in an attempt to conjure up a sense of their inner lives, their uniqueness, and to make their circumstances feel *real* to her clinical audiences (Wood, 2022, 2025).

We argue here that the photovoice method and resultant imagery aligns closely with how Saunders elaborated on her concept of ‘total pain’ in order to persuade her clinical audiences of the value of a new type of care for the dying (which eventually became the medical specialty palliative care). Just as Saunders populated her anecdotes with everyday details to conjure up the reality and the specificity of people’s end-of-life suffering, so photographs can achieve the same effect, zooming in on the fine-grained intimate details of people’s embodied experiences and revealing the particularity of their circumstances and their experience of suffering. Stacey and Liz’s photovoice, like the concept of ‘total pain’, also directs attention to the sources of suffering *beyond* the body, so often the exclusive focus of clinical encounters of short duration.

Of the four dimensions of suffering identified in Saunders’ concept of ‘total pain’—the social dimension is the one which is least commonly discussed in the clinical literature (Rowley et al., 2021). The everyday details revealed in Stacey and Liz’s photovoice images—Stacey’s close-up of her scalp, balding from the effects of chemotherapy or the screenshot of yet another unsuccessful bids on a new flat; Liz’s pink sewing machine or her shakily-written notes scribbled in the middle of the night—bring into view the unique configuration of these women’s identity, housing and material circumstances, the structural disadvantages they had experienced across the life course, as well as their emotional responses to their life coming to an end. Stacey and Liz’s photographs provide an insider’s (emic) view of their life, so often missing in palliative and end of life care (Richards et al., 2024), and bring the housing inadequacies, noise, damp, cold, and bureaucratic delays they experienced into public view, making a direct call to action (Wang, 1999: 190), not just to those whose professional role is to respond to end-of-life suffering, but to wider society. Saunders herself took photographs of her patients, and Wood (2025) devotes a chapter of his book to discussing these and interrogating the intentions behind them. Wood (2025) argues that Saunders’ aim in producing and sharing these (semi-staged) photographs of her patients “living while dying”, as she phrased it, was to de-medicalize how professionals looked at dying patients and to produce a visual record of her new form of medical care. These photographs were not intended to represent ‘total pain’, but rather its relief. The new form of care Saunders was championing, represented in the concept of ‘total pain’, was reliant on witnessing, companionship, and, most importantly, not looking away or abandoning patients to suffer alone (resulting in Saunders’ well documented use of the biblical phrase ‘watch with me’) (Clark, 2018; Wood, 2025).

However, the photographs taken by Saunders on her ward rounds were inevitably mediated by her and she retained the power over both the image-making and the interpretation of the images:

Saunders’s photographs depend on being interpreted and interpretable within the context that *she* provides, potentially in ways that conceal the interior experience of her patients (Wood, 2025: 159, *emphasis added*).

In contrast, photovoice involves researchers handing power over the image-making to the patient herself who controls *what* is shown and the interpretation of the images during the various touchpoints (Milne and Muir, 2020). This indicates a significant shift from Saunders’ era in terms of who holds the representational and interpretive power—the professional or the dying individual. One of the strengths of the photovoice method is that, unlike interview-based research methods, it does not rely on participants’ ability to articulate their experiences in words alone, or to be able to do so in the predominant language of the country (Milne and Muir, 2020). Furthermore, participants do not need to wait passively for the issues they are facing to be diagnosed by professionals but rather can reveal the insider or ‘emic’ view. Finally, in contrast to Saunders’ photographs, Stacey and Liz’s photovoice images evoke a sense of what life is like for them *outside* of institutions, where public health palliative care experts claim that 95% of dying takes place (Kellehear, 2022). If Saunders’ intended her concept of ‘total pain’ to encourage clinicians to understand the *wider context* of people’s lives and to take into account the multi-dimensional nature of end-of-life suffering when treating them, then photovoice imagery contributes to that same end, widening the field of view. However, it represents that suffering on the patient’s *own* terms.

Photovoice, however, is not a research method or ‘tool for action’ which can represent the entirety of someone’s life or their inner world. There will always be absences and exclusions—images not taken or not able to be taken—as well as what simply cannot be conveyed in an image or series of images (Milne and Muir, 2020). As Saunders herself came to understand, people’s suffering can be extremely complex making it very difficult to either articulate in words or represent in images, even when taken by the person themselves of their own experiences. Saunders tried to convey her concept of ‘total pain’ indirectly through poetic fragments or particularly evocative metaphors used by her patients, most famously the expression “all of me is wrong” used by her patient Mrs. Hinson. These fragments were either used as emblematic of the indivisibility of the various dimensions of suffering or as signals for the kind of care that was required to ameliorate such suffering:

The ‘total’ of ‘total pain’ might therefore not be something which requires an emplotted narrative but which acknowledges a level of complexity that can only be appreciated obliquely through one or more of its parts (Wood, 2022: 416).

Social researchers often try to use photovoice imagery to ‘emplot’ a narrative over time, but as Wood (2022, 2025) argues in the case of total pain, sometimes depth and complexity can only be appreciated obliquely and in fragments, not in forced narratives. We have selected images and quotes for this paper which could be accused of emplotting just such a narrative for the purpose of telling a coherent storey about our participants’ final year of life. However, our experiences of presenting these images to a variety of audiences indicate that their power lies more in the small telling details of individual images; details which do not try to explain their suffering in its entirety but rather render it palpable and resonant. Their advocacy value lies precisely in “imbuing social science analysis with emotional, documentary and aesthetic power” (Bourgois, 2011: 6)—arresting attention and making otherwise overlooked circumstances newly visible.

## Conclusion

Stacey and Liz, Dying in the Margins participants who never met one another, coincidentally died in adjacent rooms in a Glasgow in-person hospice, serving a part of the city with high rates of relative deprivation. Whilst Stacey spent the last year of her life trying to find a new home which would meet her needs, Liz spent it struggling to remain in her home of over a decade. Neither achieved their wishes. The photovoice images taken by Stacey and Liz over the last months of their lives provide an insider’s view of their end-of-life suffering—often lacking in research—and reveal, in alignment with Saunders’ concept of ‘total pain’—the situated, contextual and multiple causes of that suffering with damp, overcrowding, noise pollution, a lack of access to green spaces and so on compounding their physical pain and contributing to a whole overwhelming experience. The photovoice method was also able to capture sources of strength and of individual identity, self-advocacy, and of small moments of kindness—such as Stacey’s photograph showing the tea and biscuits provided by the “wee volunteer women” in the hospital.

The context of a patient’s wider life often lies beyond sight of clinical professionals and therefore the intersecting and compounding causes of end-of-life suffering can be missed. Photovoice can provide this insight, increasing the emotional identification which Saunders herself was seeking when she narrated fragments of patients’ storeys to illustrate her concept of ‘total pain’. Stacey and Liz chose to photograph certain details and moments because of their emblematic significance—they were directing not just the clinical gaze, but the policymaker’s gaze and public attention, making visible and *real* the multiple dimensions of their suffering to those willing to take the time to look.

## Data Availability

The original contributions presented in the study are included in the article/supplementary material, further inquiries can be directed to the corresponding author.
